# The human gut serves as a reservoir of hypervirulent *Klebsiella pneumoniae*

**DOI:** 10.1080/19490976.2022.2114739

**Published:** 2022-08-24

**Authors:** Jing Yang, Yi Li, Na Tang, Juan Li, Juan Zhou, Shan Lu, Gui Zhang, Yuqin Song, Chao Wang, Jin Zhong, Jianguo Xu, Jie Feng

**Affiliations:** a State Key Laboratory of Infectious Disease Prevention and Control, National Institute for Communicable Disease Control and Prevention, Chinese Center for Disease Control and Prevention, Beijing, China; b State Key Laboratory of Microbial Resources, Institute of Microbiology, Chinese Academy of Sciences, Beijing, China; c College of Life Science, University of Chinese Academy of Sciences, Beijing, China; d Institute of Public Health, Nankai University, Tianjin, China

**Keywords:** Hypervirulent *Klebsiella pneumoniae*, healthy people, human gut, reservoir

## Abstract

Hypervirulent *Klebsiella pneumoniae* (hvKp) can cause serious infections and has been increasingly reported clinically. However, we still lack the knowledge to what degree hvKp colonize the community. In this study, we investigated colonization of hvKp in healthy human gut and the relationship between gut hvKp and clinically important invasive strains. We compile global genomes of gut *K. pneumoniae* for in-depth genetic analysis and found most hvKp genomes originated from Chinese datasets; therefore, we collected gut *K. pneumoniae* isolates from healthy people around China. The results revealed a moderate carriage rate of hvKp in the healthy population (4%–5.19%). Phylogenetic analysis indicated a close relationship between gut hvKp and fatal clinical strains. These results demonstrate that the human gut may serve as a reservoir of hvKp and that gut hvKp can play a role in infection of other body parts.

## Introduction

*Klebsiella pneumoniae* is a member of the family Enterobacteriaceae that is best known for its capacity to cause infections, including healthcare-associated infections and community-acquired infections (CAIs). CAIs are often caused by hypervirulent *K. pneumoniae* (hvKp) and cause severe disease, with a hallmark clinical syndrome of hepatic abscess in the absence of biliary tract disease. Meanwhile, hvKp can infect other parts of the body.^1^ Serious infections caused by hvKp have been primarily observed in East Asia; however, reports of infections with hvKp have been increasing worldwide.^2^,^3^ The evolution of hvKp has been largely driven by the acquisition of virulence genes on mobile genetic elements. HvKp acquired extra siderophore system to capture iron from the host using proteins encoded by *iuc* (encoding aerobactin), and *iro* (encoding salmochelin). Hypermucoid is a phenotype commonly associated with hypervirulence and results from capsule overexpression which is regulated by two genes, *rmpA* and *rmpA2*. The two genes are generally co-located on large plasmids. Moreover, *rmpA*/*rmpA2, iuc* locus, and *iro* locus are used as biomarkers to differentiate hvKp from classical *K. pneumoniae*.^1^

Several studies have demonstrated that the human gut functions as a reservoir of *K. pneumoniae*. The prevalence of *K. pneumoniae* colonic colonization was 23.0% among patients admitted to intensive care units of one hospital in the United States, and was estimated as 6% in a similar case in Australia.^4^,^5^ Both studies showed that gut colonization on admission was significantly associated with subsequent infection. Aside from hospital patients, *K. pneumoniae* has been found in healthy humans in the community. High gut colonization has been observed in Asia, with colonization rates of 87.7%, 61.1%, 57.9%, 18.8%, 52.9%, and 41.3% in Malaysia, Singapore, China, Japan, Thailand, and Vietnam, respectively.^6^ Another study of pregnant women from communities in Madagascar, Cambodia, and Senegal reported a *K. pneumoniae* colonization rate in stool of 55.9%;^7^ the survey also found specific serotypes strongly associated with hvKp in the human gut, indicating that the human gut plays a role in storing hvKp. However, accurate data on hvKp gut colonization rates of individuals from the community and on the relationship between gut hvKp and clinical strains are lacking, and such information is essential to guiding hvKp infection prevention and control practices beyond standard precautions.

Recent developments in sequencing technology have enabled in-depth exploration of complex microbial communities and microbiomes, especially in the human gut. For this study, we first collected global human gut *K. pneumoniae* genomes, intending to describe the distribution of hvKp based on genetic annotation. Statistical analysis revealed that hvKp was present only in the dataset of Chinese gut samples. Next, a group of gastrointestinal *K. pneumoniae* strains was isolated from healthy people in Chinese cities and detailed information was obtained. We thereby revealed that the human gut functions as a reservoir of hvKp and has a significant association with clinical infections.

## Results

### Clones of hvKp are present in a gut metagenome dataset of the Chinese population

**Figure 1.**
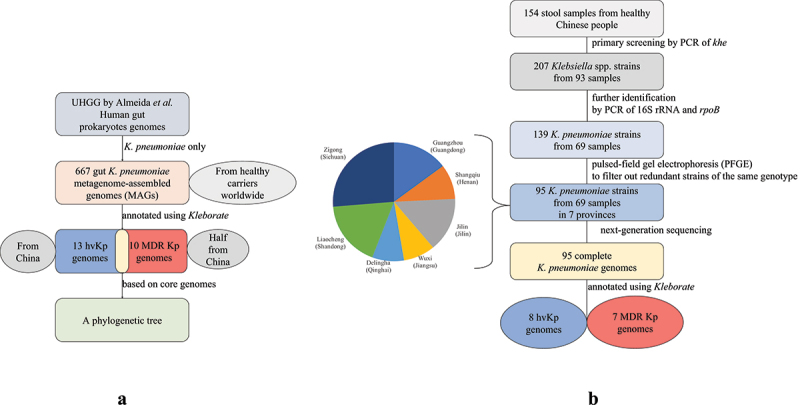
Workflow of data collection. a. The collection of global *K. pneumoniae* MAGs from online datasets. b. Isolation of *K. pneumoniae* strains from healthy Chinese population gut.

The genetic analysis of human gut *K. pneumoniae* utilized in our study was inspired by the work of Alexandre Almeida *et al*. prior to 2019.^8^ Almeida *et al*. presented the Unified Human Gastrointestinal Genome (UHGG) collection, comprising more than 200,000 genomes of 4,644 gut prokaryotes. We only took advantage of the Metagenome-assembled genomes (MAGs) of *K. pneumoniae* as further research materials (Table 1). Six hundred and sixty-seven gut *K. pneumoniae* MAGs were selected for screening hvKp and identifying its colonization rate (Table S1, Figure 1a). These data were derived from more than 64 studies conducted worldwide across five continents. Tracing back to study details helped to clarify the makeup of *K. pneumoniae* hosts. Most of the hosts were healthy carriers, from infants to seniors, with a few affected by mild chronic diseases. Overall, *K. pneumoniae* MAGs appear in the guts of healthy people worldwide. Notably, China and the United States accounted for the largest percentages of host MAG locations, with 255 (38.2%) and 281 (42.1%), respectively.

**Table 1.** Three datasets analyzed in our study and the prevalence of (hv)Kp in each.

Datasets	Data source	Sample number	Kp number	Ratio^a^	HvKp number	Ratio^b^
I	UHGG collection by Almeida *et al.*	\	667	\	13	\
II	Sample isolation from seven provinces in China	154	69	44.8%	8	5.19%
III	Sample isolation from families in two villages in China	175	30	17.14%	7	4%

*Sample number refers to the number of stool samples we took from the study cohorts, equal to the number of the cohort population.

*Ratio^a^ = the number of samples identified with *K. pneumoniae*/the number of samples in the cohort * 100%; Ratio^b^ = the number of samples identified with hvKp/the number of samples in the cohort * 100%.

**Figure 2.**
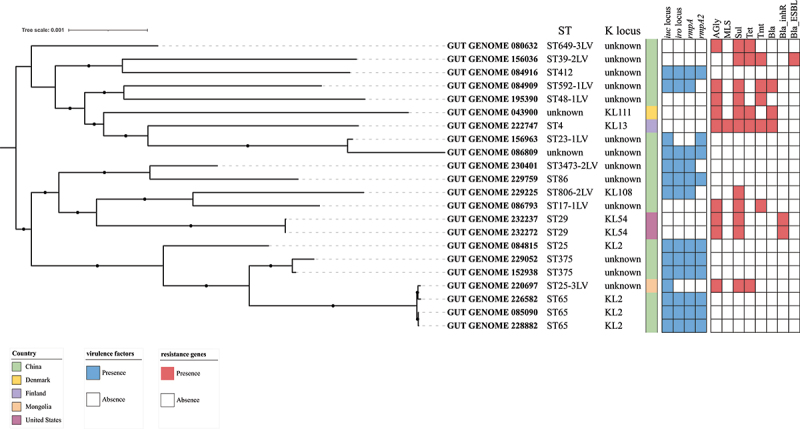
Phylogenetic tree of hypervirulent or MDR *K. pneumoniae* genomes collected from an online database with detailed characteristics attached to each genome. STs, K locus types, and the presence of genes were annotated using Kleborate ver. 2.0.4. The countries of collection are indicated with color patches.

All 667 MAGs were annotated using Kleborate version 2.0.4, providing detailed information about multilocus sequence types (STs), K locus types, virulence genes, and antimicrobial resistance genes (Table S2). Known hypervirulent and multiple-drug-resistant (MDR) *K. pneumoniae* were quite separated in the study population. In other words, gut microbiota containing both hypervirulent and MDR *K. pneumoniae* were barely detected. Thirteen MAGs (1.95%) were identified as hypervirulent and 10 (1.48%) were identified as MDR from the 667 total MAGs, with only one genome that was identified as both hypervirulent and MDR (Table S3). Based on the core genomes of those 22 target *K. pneumoniae*, we constructed a detailed phylogenetic tree (Figure 2). Two clades with 98% bootstrap support were identified, and the distribution of hypervirulent and MDR *K. pneumoniae* in those two clades indicated the phylogenetic diversity of these strains. The hvKp strains included ST65, ST375, ST25, ST806, ST86, ST3473, ST412, ST23, and ST592. Of the 13 hypervirulent strains, nine exhibited the genetic pattern of *iuc, rmpA, rmpA2* plus *iro*, while three strains lacked the gene *rmpA2*. Only one strain exhibited the pattern of *iuc* and *rmpA2*. The capsule locus types of more than half of hvKp strains were not clearly identified. The identified K locus types consisted of 80% (four strains) KL2 and 20% (one strain) KL108. Notably, with the exception of one strain, all hypervirulent strains lacked detected resistance genes. The STs and K locus types of MDR strains were diverse. However, no prevalent MDR clones that frequently cause nosocomial infections were detected. Although 10 MAGs carried antimicrobial resistance (AMR) genes against various drugs, none carried antimicrobial resistance genes associated with carbapenem resistance.

When considering collection countries, we noted high abundance of hvKp in gut samples from the Chinese population. All hypervirulent MAGs were detected in the Chinese dataset and accounted for 5.1% of all Chinese gut *K. pneumoniae* carriers, suggesting that the healthy population in China is likely to carry hvKp in their guts. Overall, five out of ten MDR *K. pneumoniae* MAGs were found in the Chinese population, while the other five were scattered among Denmark, Finland, and Mongolia, suggesting that resistance genes have spread in the human gut across multiple countries.

### K. pneumoniae *isolates collected in China reveal the genetic patterns of the gut microbiota in healthy people*

To obtain more detailed information about gut *K. pneumoniae* in the Chinese population, a total of 154 stool samples from healthy people were used for isolation of *K. pneumoniae*. We collected 95 gut *K. pneumoniae* isolates from 69 healthy people living in urban and rural areas of seven Chinese provinces (Table 1). These 69 *K. pneumoniae* carriers correspond to a prevalence of 44.8%. The whole population consisted of 33 female and 36 male participants, with ages evenly distributed from 18 to 59. Urban and rural sources contributed 38 and 31 samples, respectively, which were collected in all seven provinces in China (Table S4). Next-generation sequencing and assembly produced 95 *K. pneumoniae* genomes, which were later processed and annotated using Kleborate. Overall, 72 STs and 55 K locus types were found, including ST17, ST101, ST1966, ST23, ST277, ST432, KL107, KL1, KL2, and KL46, which were scattered throughout the population (Table S5). As noted above, the hypervirulent and MDR-related MAGs in the Chinese population accounted for 5.10% and 1.96%, respectively, of the 255 total Chinese gut *K. pneumoniae* MAGs. Among the 95 isolates in our collection, eight and seven isolates were identified as hypervirulent and MDR *K. pneumoniae*, respectively (Figure 1b). Notably, each of these isolates was obtained from a different sample. The hvKp carriage rate was 11.59% and the MDR *K. pneumoniae* prevalence was 10.14% among 69 healthy *K. pneumoniae* carriers. The carriage rates in our collection were higher than those in previous global studies. The hvKp detection rates in this population (154 individuals) was as high as 5.19%. The 15 target samples, regardless of virulence or resistance, were derived from diverse regional backgrounds and human sources, including urban and rural areas in six cities and ages from 18 to 53, with no clustering based on region, gender, or age.

The phylogenetic tree based on the core genomes of these isolates showed no significant clustering of hypervirulent or MDR *K. pneumoniae* isolates (Figure S1). According to genomic annotation, the eight hypervirulent isolates showed slight connections with ST23 and KL1 and KL2. The hypervirulent isolates included three ST23 strains, along with several individual strains (ST412, ST268, ST65, ST380, and ST1265). Among the hypervirulent group, four KL1 and two KL2 types were detected, along with KL57 and KL20 strains. The same three ST23 strains were identified as having the K locus type KL1. This consistency did not carry over to their geographic distribution. The strains originated from different provinces, Shandong, Jilin, and Guangdong, despite their close relatedness. In general, the eight hypervirulent isolates were evenly distributed among four provinces, Shandong, Jilin, Qinghai, and Guangdong. The virulence genetic patterns were similar to previous results reported worldwide. Of eight hypervirulent strains, six carried all four virulence genes (*iuc, rmpA, rmpA2* plus *iro*), while one strain lacked *rmpA2*. Only one strain showed the pattern of *iuc* and *rmpA2*. None of the hypervirulent strains exhibited antimicrobial resistance. Notably, all gut hvKp in our collection showed STs of prevalent hypervirulent clones that cause severe infections.

The seven MDR *K. pneumoniae* isolates were identified as containing non-overlapping STs (ST4467, ST261, ST716, ST1940, ST101, ST111, and ST497) and sharing no common STs. In addition, none of the MDR isolates carried carbapenem-resistance genes while most of them carried the genes conferring resistance to aminoglycosides, fluoroquinolones, sulfonamides, tetracyclines. This finding was in accordance with the previous result of our global screening on MDR strain carriers that none of those 10 MDR strains investigated contained carbapenem-resistance genes. This result was unexpected, as the Chinese clinical data indicate that ST11 carbapenem-resistant *K. pneumoniae* (CRKp) has been a prevalent AMR *K. pneumoniae* clone in Chinese hospitals. This discrepancy suggests that the human gut may function as a reservoir of hvKp rather than carbapenem-resistant clones in the community. Notably, most MDR *K. pneumoniae* isolates were collected in urban areas, while hypervirulence exhibited no regional specificity, which might be associated with more frequent antimicrobial application in urban areas (Table S4).

The dataset above included the distribution patterns of hvKp strains in the gut of healthy individuals from different provinces of China. We also screened healthy populations for gut hvKp strains in two rural locales. We collected samples from two villages 15 km apart (referred as A and B) in Wuxi, Jiangsu, China in 2017 (Table 1). In village A, 87 stool samples were collected from 87 healthy villagers from 45 families. Twelve Kp strains were isolated, two of which being subsequently identified as hvKp. Thus, the proportion of hvKp in all *K. pneumoniae* isolates was approximately 16.67% and the hvKp detection rate in this population was 2.3%. In village B, 88 stool samples were collected from 88 healthy villagers from 43 families. Eighteen Kp strains were isolated and five of them were identified as hvKp. The proportion of hvKp in all *K. pneumoniae* isolates was thus about 27.78% and the hvKp detection rate in this population was 5.68%. There was no significant difference between these two villages in the distribution of hvKp, both in strains and population. The identified hvKp varied in their STs and serotypes. The STs and serotypes included ST36, ST23, ST2165, ST375, ST 660 and KL62, KL1, KL2, KL16. No cases had been found wherein more than one member of the same family carried hvKp simultaneously.

### The genomic analysis reveals that hvKp strains in the gut are phylogenetically closely related to strains known to be implicated in clinical infections

With potentially infectious pathogens in human gut, determining the degree to which gut colonization hvKp in healthy people may develop into infection is an urgent task. We first downloaded nearly all (11,568) *K. pneumoniae* genome assemblies from the National Center for Biotechnology Information (NCBI), which are derived from various isolation sources and geographic locations. A huge phylogenetic tree was constructed from the core genomes of the 22 hv/MDR Kp MAGs identified from the global gut *K. pneumoniae* database, 95 isolates from healthy Chinese population gut in our collection, and more than 10,000 genomes recorded in NCBI. Our focus was on strains phylogenetically close to *K. pneumoniae* in healthy human gut; therefore, we ignored all other irrelevant branches and retained only the minimum set of branches (branch length < 0.003) containing our defined gut *K. pneumoniae* strains. As shown in Figure S2, each small branch represented one specific consistent ST that clustered phylogenetically close genomes. Most of the gut *K. pneumoniae* strains (97/117) had relatives in the NCBI database, which allowed us to further investigate their genetic backgrounds and relationships with clinical strains. It was calculated that the number of SNPs between defined gut hvKp and those clinical strains was mostly under 1000 (71.64%) and characterized with the number between 118 and 520. First, all 8 hvKp isolates in our collection found their relatives which were mainly associated with clinical infection diseases, such as bloodstream infections, liver abscess, respiratory tract infections, urinary tract infections, and wound infections. 12 out of 13 hvKp genomes in MAGs also found their relatives which were isolates from various infections. However, the non-hvKp in human gut was found less connection with clinical infections. Three out of the seven MDR strains isolated could not find their relatives. Among the 80 strains without virulence factors or resistance genes, 15 could not find their relatives while 20 had relatives irrelevant to clinical infections.

In general, nearly every gut hvKp MAG or isolate in our research was associated with clinical counterparts either in China or overseas. The similar genetic backgrounds of gut hvKp and clinical strains indicate that roughly the same *K. pneumoniae* strains cause infections in some parts of the body while also hiding in the healthy human gut.

## Discussion

Studies have demonstrated that *K. pneumoniae* is a common component of the human gut microbiome and that its carriage rate varies among populations. However, data on the reservoirs and mechanism of spread of hvKp strains is lacking, as hvKp-specific markers have not been consistently applied to determine their relative proportion among *K. pneumoniae*. In this study, we analyzed data available from public databases and found that hvKp can colonize the guts of Chinese people. Then, we collected *K. pneumoniae* from healthy Chinese people’s guts, which included hvKp. This result was consistent with the results obtained from public databases, but with a higher carriage rate. Colonization of the gut by hvKp has also been observed in other countries. A recent study reported the presence of hvKp in the guts of pregnant women in Madagascar and Cambodia, but the rate of strains co-carrying siderophores and capsule regulators was very low.^7^ In addition, 5 *K. pneumoniae* strains were defined as hypervirulent among 484 *K. pneumoniae* strains isolated from fecal samples from an adult population in Norway.^9^ Our findings indicate that the human gut, in particular in the Chinese population, can serve as a major reservoir of hvKp. Understanding the reservoirs of hvKp is essential to preventing and controlling the dissemination of this dangerous pathogen.

Infection with hvKp can lead to liver abscess. A study of patients with liver abscesses in Taiwan demonstrated a strong relationship between gastrointestinal carriage of hvKp and liver abscesses.^10^ Infections caused by hvKp have been frequently reported in Asia, especially in China, and the prevalence of hvKp isolates in the gut may be related to the prevalence of hvKp infections in China. Furthermore, all STs with specific serotype found in the healthy gut had been reported to cause CAIs (Table S8). ST23 with serotype KL1 isolates were found in three samples, belonging to the clonal group CG23. CG23 has been shown to account for the majority of *K. pneumoniae* liver abscesses in Asia.^11^ From these results, combined with the close phylogenetic relationship between gut and clinical hvKp, a linkage between the gut *K. pneumoniae* reservoir in healthy people and clinical infections can be inferred. We therefore hypothesize that in the healthy human gut, hvKp carriage is an unstable factor that may develop into invasive infections resulting in disease.

CRKp represents a serious public health threat worldwide. The most prevalent CRKp clonal group is CG258, consisting of three STs: ST258, ST11, and ST512.^12^ ST11 is the predominant clone in Asia, especially in China.^13^ However, no CG258 *K. pneumoniae* strain was detected, and no strain was found to harbor carbapenem resistance genes in our collection, indicating that gastrointestinal carriage is not a major reservoir of CRKp in individuals in community. However, several studies have shown that the gut isolates of CRKp in hospitalized patients are closely related to the successive infection,^14^ suggesting that the gut could also be the reservoir of CRKp in patients with recent healthcare contact. The convergence of carbapenem resistance genes and virulence factors on the same or coexisting plasmids can result in the development of CR-hvKp, which is approaching the worst-case scenario.^15^ Carbapenem-resistant hvKp (CR-hvKp) has been reported to cause fatal nosocomial infections in China.^16^ However, no strain in our collection carries carbapenem resistance genes, suggesting that the healthy human gut is not a hot spot of convergence for carbapenem-resistance and virulence genes.

A limitation of this study was the small sample size. Regardless, our results provide insights into the carriage rate of hvKp, MDR Kp in the heathy population and their reservoirs and demonstrate the need for further studies on hvKp colonization.

## Methods

### *Isolation of* K. pneumoniae

A total of 154 stool samples from healthy people were used for the isolation of *K. pneumoniae* in this study. Volunteer recruitment and sample collection were described in detail in a previous study.^17^ We took 2 g of stool sample, enriched it with 5 mL of GN broth (Cat: CM218; Beijing Land Bridge Tech, Beijing, China) and incubated the resulting mixture at 37°C for 18–24 h on a shaker (220 rpm). For each enrichment sample, 1 μL was centrifuged at 12,000 × *g* for 2 min to harvest the pellet, which was then suspended in 100 μL of lysis buffer (100 mM NaCl, 10 mM Tris–HCl [pH 8.3], 1 mM EDTA [pH 9.0], and 1% Triton X-100), boiled for 10 min, and centrifuged. The resulting supernatant was used as a template to test for the presence of the *khe* gene (specific to the genus *Klebsiella*) using PCR with specific primers (khe-F: TGATTGCATTCGCCACTGG; khe-R: GGTCAACCCAACGATCCTG; the target product length is 428 bp^18^,^19^). One loopful (10 μL) of each enrichment culture (whether *khe*-positive or not) was directly streaked onto MacConkey agar (Cat: CM1169B; Oxoid, Basingstoke, UK). After overnight incubation at 37°C, pink, round, moist, and mucoid colonies, presumptively of *Klebsiella* spp., were picked and purified to test for the presence of the *khe* gene. In total, 207 *Klebsiella* spp. strains were obtained from 93 samples (*khe*-positive enrichments: 83). Further identification of *K. pneumoniae* strains was conducted through amplification of the 16S rRNA gene (primers: 27 F/1492 R) and *rpoB* gene.^20^,^21^ The resulting 139 *K. pneumoniae* strains isolated from 69 samples were digested with *Xba*I and separated through pulsed-field gel electrophoresis (PFGE) according to the protocol of PulseNet.^22^ Finally, 95 isolates of *K. pneumoniae* were selected according to their PFGE gel patterns and sequenced on the Illumina platform.

The identification of *K. pneumoniae* strains isolated from the stool samples collected in village A and B were as above. The presence of virulence genes – *iuc* locus, *rmpA*, and *rmpA2*–were detected by PCR. Those with positive PCR results of *iuc* locus and *rmpA*/*rmpA2* genes were identified as hvKp. The primers of the virulence factors were presented in Table S7.

### Genomic sequencing and bioinformatics analyses

We used the available dataset of the Unified Human Gastrointestinal Genome collection reconstructed by Almeida et al. to identify *K. pneumoniae* in the human gut microbiome.^8^ A total of 11,568 *K. pneumoniae* genomes were downloaded from the NCBI Reference Sequence Database (https://www.ncbi.nlm.nih.gov/refseq/) on September 8, 2021. Data for the isolation sources and geographic locations of these genomes were extracted from their GenBank files.

The draft genomes of 95 isolates of *K. pneumoniae* and seven hvKp isolates from village A and B were obtained on the Illumina HiSeq^TM^ 2000 platform. After filtering out low-quality reads, the clean data were *de novo* assembled into a number of scaffolds using SOAP *de novo* (http://soap.genomics.org.cn/soapdenovo.html). All genomes were annotated with GeneMarkS (http://topaz.gatech.edu/). Kleborate version 2.0.4 was used to predict genomic features, including virulence factors, antibiotic resistance loci, STs, and capsular serotypes of the genome assemblies.^23^ Phylogenetic analysis based on the core genes was conducted as described previously.^24^ We used Prokka version 1.14.5 to annotate the genomes of all strains.^25^ The General Feature Format files produced by Prokka were used as inputs for Roary version 3.13.0 with the default parameters for core gene alignment.^26^ The resulting alignment was used to infer a maximum likelihood phylogeny with FastTree ver. 2.1.10.^27^ All phylogenetic trees were visualized using Interactive Tree Of Life software.^28^ The subtree was extracted with the ETE3 toolkit.

## Supplementary Material

Supplemental Material

## Accession numbers

The genomic sequencing data of 95 isolates were deposited in the NCBI database and are accessible via the BioProject PRJNA796162. The draft genomes of seven hvKP from village A and B were deposited in the NCBI Genbank database under accession number PRJNA847686.

## References

[1] Russo TA, Marr CM. Hypervirulent *Klebsiella pneumoniae*. Clin Microbiol Rev. 2019;32(3). doi:10.1128/cmr.00001-19. PMC6589860 31092506

[2] Lin YT, Chen TL, Siu LK, Hsu SF, Fung CP. Clinical and microbiological characteristics of community-acquired thoracic empyema or complicated parapneumonic effusion caused by *Klebsiella pneumoniae* in Taiwan. Eur J Clin Microbiol Infect Dis. 2010;29(8):1003–9. doi:10.1007/s10096-010-0961-8. 20505967

[3] Lee CR, Lee JH, Park KS, Jeon JH, Kim YB, Cha C-J, Jeong BC, Lee SH. *Antimicrobial resistance of hypervirulent Klebsiella pneumoniae*: epidemiology, hypervirulence-associated determinants, and resistance mechanisms. Front Cell Infect Microbiol. 2017;7:483. doi:10.3389/fcimb.2017.00483. 29209595 PMC5702448

[4] Martin RM, Cao J, Brisse S, Passet V, Wu W, Zhao L, Malani PN, Rao K, Bachman MA. Molecular epidemiology of colonizing and infecting isolates of *Klebsiella pneumoniae*. mSphere. 2016;1(5). doi:10.1128/mSphere.00261-16 PMC5071533 27777984

[5] Gorrie CL, Mirčeta M, Wick RR, Edwards DJ, Thomson NR, Strugnell RA, Pratt NF, Garlick JS, Watson KM, Pilcher DV, et al. Gastrointestinal carriage is a major reservoir of *Klebsiella pneumoniae* infection in intensive care patients. Clin Infect Dis. 2017;65(2):208–215. doi:10.1093/cid/cix270. 28369261 PMC5850561

[6] Lin YT, Siu LK, Lin J-C, Chen T-L, Tseng C-P, Yeh K-M, Chang F-Y, Fung C-P. *Seroepidemiology of Klebsiella pneumoniae* colonizing the intestinal tract of healthy Chinese and overseas Chinese adults in Asian countries. BMC Microbiol. 2012;12(1):13. doi:10.1186/1471-2180-12-13. 22260182 PMC3273430

[7] Huynh BT, Passet V, Rakotondrasoa A, Diallo T, Kerleguer A, Hennart M, Lauzanne AD, Herindrainy P, Seck A, Bercion R, et al. *Klebsiella pneumoniae* carriage in low-income countries: antimicrobial resistance, genomic diversity and risk factors. Gut Microbes. 2020;11(5):1287–1299. doi:10.1080/19490976.2020.1748257. 32404021 PMC7527070

[8] Almeida A, Nayfach S, Boland M, Strozzi F, Beracochea M, Shi ZJ, Pollard KS, Sakharova E, Parks DH, Hugenholtz P Segata N, et al. A unified catalog of 204,938 reference genomes from the human gut microbiome. Nat Biotechnol. 2021;39(1):105–114. doi:10.1038/s41587-020-0603-3. 32690973 PMC7801254

[9] Raffelsberger N, Hetland MAK, Svendsen K, Småbrekke L, Löhr IH, Andreassen LLE, Brisse S, Holt KE, Sundsfjord A, Samuelsen Ø, et al. Gastrointestinal carriage of *Klebsiella pneumoniae* in a general adult population: a cross-sectional study of risk factors and bacterial genomic diversity. Gut Microbes. 2021;13(1):1939599. doi:10.1080/19490976.2021.1939599. 34182896 PMC8244762

[10] Fung CP, Lin Y-T, Lin J-C, Chen T-L, Yeh K-M, Chang F-Y, Chuang H-C, Wu H-S, Tseng C-P, Siu LK, et al. *Klebsiella pneumoniae* in gastrointestinal tract and pyogenic liver abscess. Emerg Infect Dis. 2012;18(8):1322–1325. doi:10.3201/eid1808.111053. 22840473 PMC3414011

[11] Lam MMC, Wyres KL, Duchêne S, Wick RR, Judd LM, Gan Y-H, Hoh C-H, Archuleta S, Molton JS, Kalimuddin S, et al. Population genomics of hypervirulent *Klebsiella pneumoniae* clonal-group 23 reveals early emergence and rapid global dissemination. Nat Commun. 2018;9(1):2703. doi:10.1038/s41467-018-05114-7. 30006589 PMC6045662

[12] Navon-Venezia S, Kondratyeva K, Carattoli A. *Klebsiella pneumoniae*: a major worldwide source and shuttle for antibiotic resistance. FEMS Microbiol Rev. 2017;41(3):252–275. doi:10.1093/femsre/fux013. 28521338

[13] Munoz-Price LS, Poirel L, Bonomo RA, Schwaber MJ, Daikos GL, Cormican M, Cornaglia G, Garau J, Gniadkowski M, Hayden MK, et al. Clinical epidemiology of the global expansion of *Klebsiella pneumoniae* carbapenemases. Lancet Infect Dis. 2013;13(9):785–796. doi:10.1016/s1473-3099(13)70190-7. 23969216 PMC4673667

[14] Wiener-Well Y, Rudensky B, Yinnon AM, Kopuit P, Schlesinger Y, Broide E, Lachish T, Raveh D. *Carriage rate of carbapenem-resistant Klebsiella pneumoniae* in hospitalised patients during a national outbreak. J Hosp Infect. 2010;74(4):344–349. doi:10.1016/j.jhin.2009.07.022. 19783067

[15] Zhang R, Lin D, Chan EWC, Gu D, Chen G-X, Chen S. *Emergence of carbapenem-resistant serotype K1 hypervirulent Klebsiella pneumoniae* strains in China. Antimicrob Agents Chemother. 2016;60(1):709–711. doi:10.1128/aac.02173-15. 26574010 PMC4704206

[16] Gu D, Dong N, Zheng Z, Lin D, Huang M, Wang L, Chan EWC, Shu L, Yu J, Zhang R, et al. A fatal outbreak of ST11 carbapenem-resistant hypervirulent *Klebsiella pneumoniae* in a Chinese hospital: a molecular epidemiological study. Lancet Infect Dis. 2018;18(1):37–46. doi:10.1016/s1473-3099(17)30489-9. 28864030

[17] Yang J, Pu J, Lu S, Bai X, Wu Y, Jin D, Cheng Y, Zhang G, Zhu W, Luo X, et al. Species-level analysis of human gut microbiota with metataxonomics. Front Microbiol. 2020;11:2029. doi:10.3389/fmicb.2020.02029. 32983030 PMC7479098

[18] Chen Z, Liu M, Cui Y, Wang L, Zhang Y, Qiu J, Yang R, Liu C, Zhou D. A novel PCR-based genotyping scheme for clinical *Klebsiella pneumoniae*. Future Microbiol. 2014;9(1):21–32. doi:10.2217/fmb.13.137. 24328378

[19] Yin-Ching C, Jer-Horng S, Ching-Nan L, Ming-Chung C. Cloning of a gene encoding a unique haemolysin from *Klebsiella pneumoniae* and its potential use as a species-specific gene probe. Microb Pathog. 2002;33(1):1–6. doi:10.1006/mpat.2002.0499. 12127794

[20] Diancourt L, Passet V, Verhoef J, Grimont PA, Brisse S. Multilocus sequence typing of *Klebsiella pneumoniae* nosocomial isolates. J Clin Microbiol. 2005;43(8):4178–4182. doi:10.1128/jcm.43.8.4178-4182.2005. 16081970 PMC1233940

[21] Mollet C, Drancourt M, Raoult D. rpoB sequence analysis as a novel basis for bacterial identification. Mol Microbiol. 1997;26(5):1005–1011. doi:10.1046/j.1365-2958.1997.6382009.x. 9426137

[22] Tenover FC, Arbeit RD, Goering RV, Mickelsen PA, Murray BE, Persing DH, Swaminathan B. Interpreting chromosomal DNA restriction patterns produced by pulsed-field gel electrophoresis: criteria for bacterial strain typing. J Clin Microbiol. 1995;33(9):2233–2239. doi:10.1128/jcm.33.9.2233-2239.1995. 7494007 PMC228385

[23] Lam MMC, Wick RR, Watts SC, Cerdeira LT, Wyres KL, Holt KE. *A genomic surveillance framework and genotyping tool for Klebsiella pneumoniae* and its related species complex. Nat Commun. 2021;12(1):4188. doi:10.1038/s41467-021-24448-3. 34234121 PMC8263825

[24] Zhang C, Ju Y, Tang N, Li Y, Zhang G, Song Y, Fang H, Yang L, Feng J. Systematic analysis of supervised machine learning as an effective approach to predicate β-lactam resistance phenotype in streptococcus pneumoniae. Brief Bioinform. 2020;21(4):1347–1355. doi:10.1093/bib/bbz056. 31192359

[25] Seemann T. Prokka: rapid prokaryotic genome annotation. Bioinformatics. 2014;30(14):2068–2069. doi:10.1093/bioinformatics/btu153. 24642063

[26] Page AJ, Cummins CA, Hunt M, Wong VK, Reuter S, Holden MTG, Fookes M, Falush D, Keane JA, Parkhill J, et al. Roary: rapid large-scale prokaryote pan genome analysis. Bioinformatics. 2015;31(22):3691–3693. doi:10.1093/bioinformatics/btv421. 26198102 PMC4817141

[27] Price MN, Dehal PS, Arkin AP, Poon AFY. FastTree 2–approximately maximum-likelihood trees for large alignments. PLoS One. 2010;5(3):e9490. doi:10.1371/journal.pone.0009490. 20224823 PMC2835736

[28] Letunic I, Bork P. Interactive tree of life (iTOL) v5: an online tool for phylogenetic tree display and annotation. Nucleic Acids Res. 2021;49(W1):W293–w296. doi:10.1093/nar/gkab301. 33885785 PMC8265157

