# Supplementary material for: The human gut serves as a reservoir of hypervirulent Klebsiella pneumoniae
Source: Gut Microbes. 2022 Aug 24;14(1):2114739. doi: 10.1080/19490976.2022.2114739 (PMC9415575; doi:10.1080/19490976.2022.2114739)
Supplement: Supplemental Material [file KGMI_A_2114739_SM6750.zip › Supplementary Table 8.docx]

| **Supplementary Table 8. The reported infections by *K. pneumoniae* with different ST and serotype combinations** | | | |
| --- | --- | --- | --- |
| **Strain** | **ST** | **K_locus** | **Infections reported** |
| GUT_GENOME084815 | ST25 | KL2 | bacteraemia in a teaching hospital in Spain^1^  an outbreak in a high-complexity hospital from Ecuador^2^ |
| GUT_GENOME085090 GUT_GENOME226582 GUT_GENOME228882 Kp.077 | ST65 | KL2 | various invasive infections in Wenzhou, east China^3^ bacteremia in Shenzhen and Shanghai, China^4^ causing surgical site infections in China^5^ |
| GUT_GENOME229225 | ST806-2LV | KL108 | None |
| Kp.008 Kp.069 Kp.104 | ST23 | KL1 | severe and fatal infections (liver abscess) in France^6^ ventilator-associated pneumonia with high mortality rate in Iran^7^ lethal ventilator-associated pneumonia in the elderly in China^8^ pyogenic liver abscess in Portugal^9^ pneumonia and liver abscess among pediatric populations in Beijing^10^ |
| Kp.040 | ST268 | KL20 | severe systemic infections with fatal incomes in the neuro-ICU^11^ |
| Kp.048 | ST380 | KL2 | severe and fatal infections (rapidly fatal bacteremia) in France^6^ disseminated infection with a fatal outcome in a Caucasian man^12^ the first multifocal invasive infections due to *K. pneumoniae* observed in Switzerland^13^ |
| Kp.107 | ST1265-1LV | KL1 | severe acute pancreatitis in China^14^ |
| Kp.019 | ST412 | KL57 | causing bloodstream infections in Beijing, China^15^ |

**Reference**

1 Cubero, M. *et al.* Hypervirulent *Klebsiella pneumoniae* clones causing bacteraemia in adults in a teaching hospital in Barcelona, Spain (2007-2013). *Clinical Microbiology and Infection* **22**, 154-160, doi:10.1016/j.cmi.2015.09.025 (2016).

2 Reyes, J. *et al.* Characterization of bla(KPC-2): Harboring *Klebsiella pneumoniae* isolates and mobile genetic elements from outbreaks in a hospital in Ecuador. *Microbial Drug Resistance* **27**, 752-759, doi:10.1089/mdr.2019.0433 (2021).

3 Guo, Y. *et al.* Microbiological and clinical characteristics of hypermucoviscous *Klebsiella pneumoniae* isolates associated with invasive infections in China. *Frontiers in Cellular and Infection Microbiology* **7**, doi:10.3389/fcimb.2017.00024 (2017).

4 Zheng, J.-x. *et al.* Biofilm formation in *Klebsiella pneumoniae* bacteremia strains was found to be associated with CC23 and the presence of wcaG. *Frontiers in Cellular and Infection Microbiology* **8**, doi:10.3389/fcimb.2018.00021 (2018).

5 Zhao, Q., Guo, L., Wang, L.-F., Zhao, Q. & Shen, D.-X. Prevalence and characteristics of surgical site hypervirulent *Klebsiella pneumoniae* isolates. *Journal of Clinical Laboratory Analysis* **34**, doi:10.1002/jcla.23364 (2020).

6 Decre, D. *et al.* Emerging severe and fatal infections due to *Klebsiella pneumoniae* in two university hospitals in France. *Journal of Clinical Microbiology* **49**, 3012-3014, doi:10.1128/jcm.00676-11 (2011).

7 Tabrizi, A. M. A., Badmasti, F., Shahcheraghi, F. & Azizi, O. Outbreak of hypervirulent *Klebsiella pneumoniae* harbouring bla(VIM-2) among mechanically-ventilated drug-poisoning patients with high mortality rate in Iran. *Journal of Global Antimicrobial Resistance* **15**, 93-98, doi:10.1016/j.jgar.2018.06.020 (2018).

8 Liu, C. & Guo, J. Characteristics of ventilator-associated pneumonia due to hypervirulent *Klebsiella pneumoniae* genotype in genetic background for the elderly in two tertiary hospitals in China. *Antimicrobial Resistance and Infection Control* **7**, doi:10.1186/s13756-018-0371-8 (2018).

9 Pereira, A., Petrucci, T. & Simoes, M. J. *Klebsiella pneumoniae* from K1 and hypervirulent clone ST23: first documented case in Portugal. *Acta Medica Portuguesa* **30**, 496-499, doi:10.20344/amp.7705 (2017).

10 Li, Y. *et al.* Hypervirulent *Klebsiella pneumoniae* infections in pediatric populations in Beijing (2017-2019) Clinical characteristics, molecular epidemiology and antimicrobial susceptibility. *Pediatric Infectious Disease Journal* **40**, 1059-1063, doi:10.1097/inf.0000000000003253 (2021).

11 Fursova, N. K. *et al.* Multidrug-resistant *Klebsiella pneumoniae* causing severe infections in the neuro-ICU. *Antibiotics-Basel* **10**, doi:10.3390/antibiotics10080979 (2021).

12 Hentzien, M. *et al.* Seven hypervirulent ST380 *Klebsiella pneumoniae* septic localizations. *Medecine Et Maladies Infectieuses* **47**, 171-173, doi:10.1016/j.medmal.2016.10.002 (2017).

13 Flury, B. B., Dona, V., Buetti, N., Furrer, H. & Endimiani, A. First two cases of severe multifocal infections caused by *Klebsiella pneumoniae* in Switzerland: characterization of an atypical non-K1/K2-serotype strain causing liver abscess and endocarditis. *Journal of Global Antimicrobial Resistance* **10**, 165-170, doi:10.1016/j.jgar.2017.04.006 (2017).

14 Li, C. D. *et al.* A rare carbapenem-resistant hypervirulent K1/ST1265 *Klebsiella pneumoniae* with an untypeable bla(KPC)-harboured conjugative plasmid. *Journal of Global Antimicrobial Resistance* **22**, 426-433, doi:10.1016/j.jgar.2020.04.009 (2020).

15 Ma, Y. *et al.* Microbiological characterisation of *Klebsiella pneumoniae* isolates causing bloodstream infections from five tertiary hospitals in Beijing, China. *Journal of Global Antimicrobial Resistance* **12**, 162-166, doi:10.1016/j.jgar.2017.10.002 (2018).
