# Supplementary figures and images for: The human gut serves as a reservoir of hypervirulent Klebsiella pneumoniae
Source: Gut Microbes. 2022 Aug 24;14(1):2114739. doi: 10.1080/19490976.2022.2114739 (PMC9415575; doi:10.1080/19490976.2022.2114739)

Tree scale: 0.01

virulence phenotype

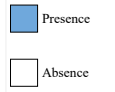

MDR phenotype

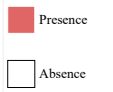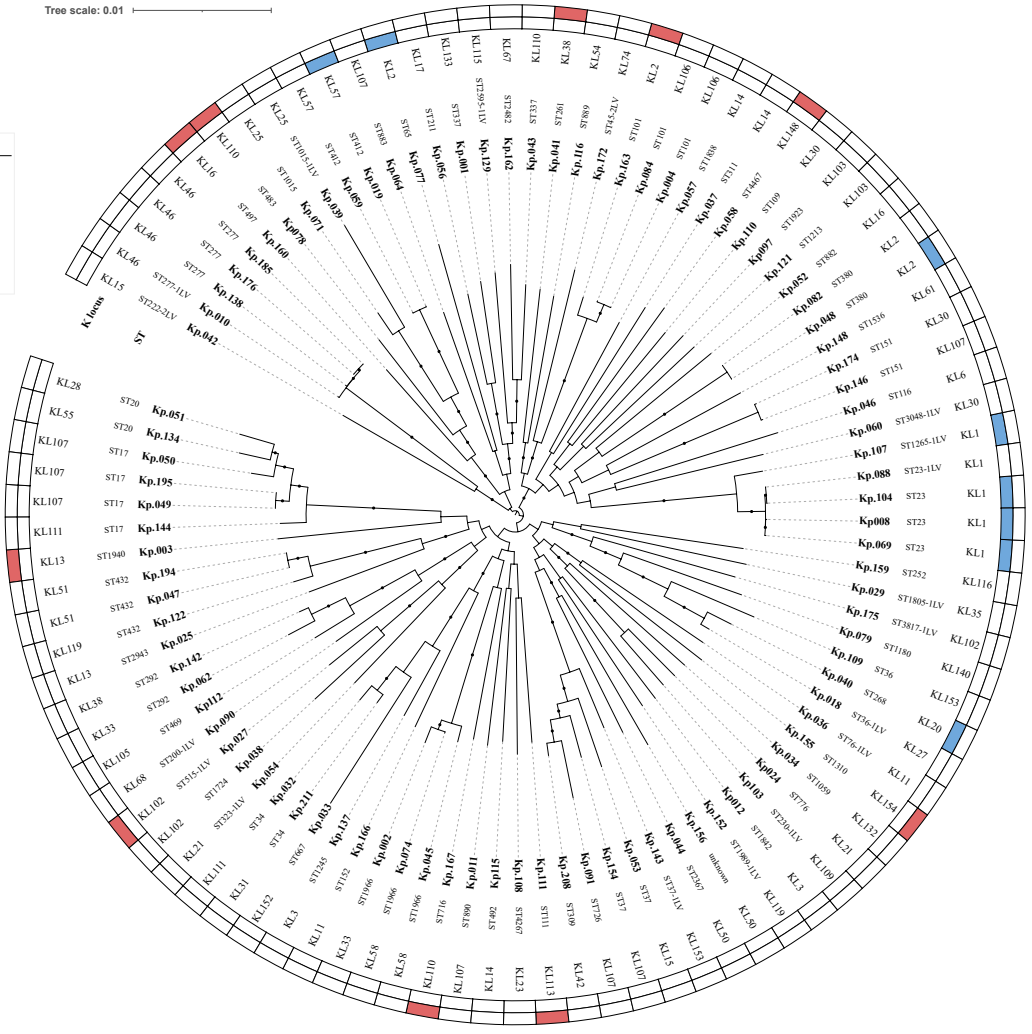

Supplement: Supplemental Material [file KGMI_A_2114739_SM6750.zip › Figure S1.pdf]

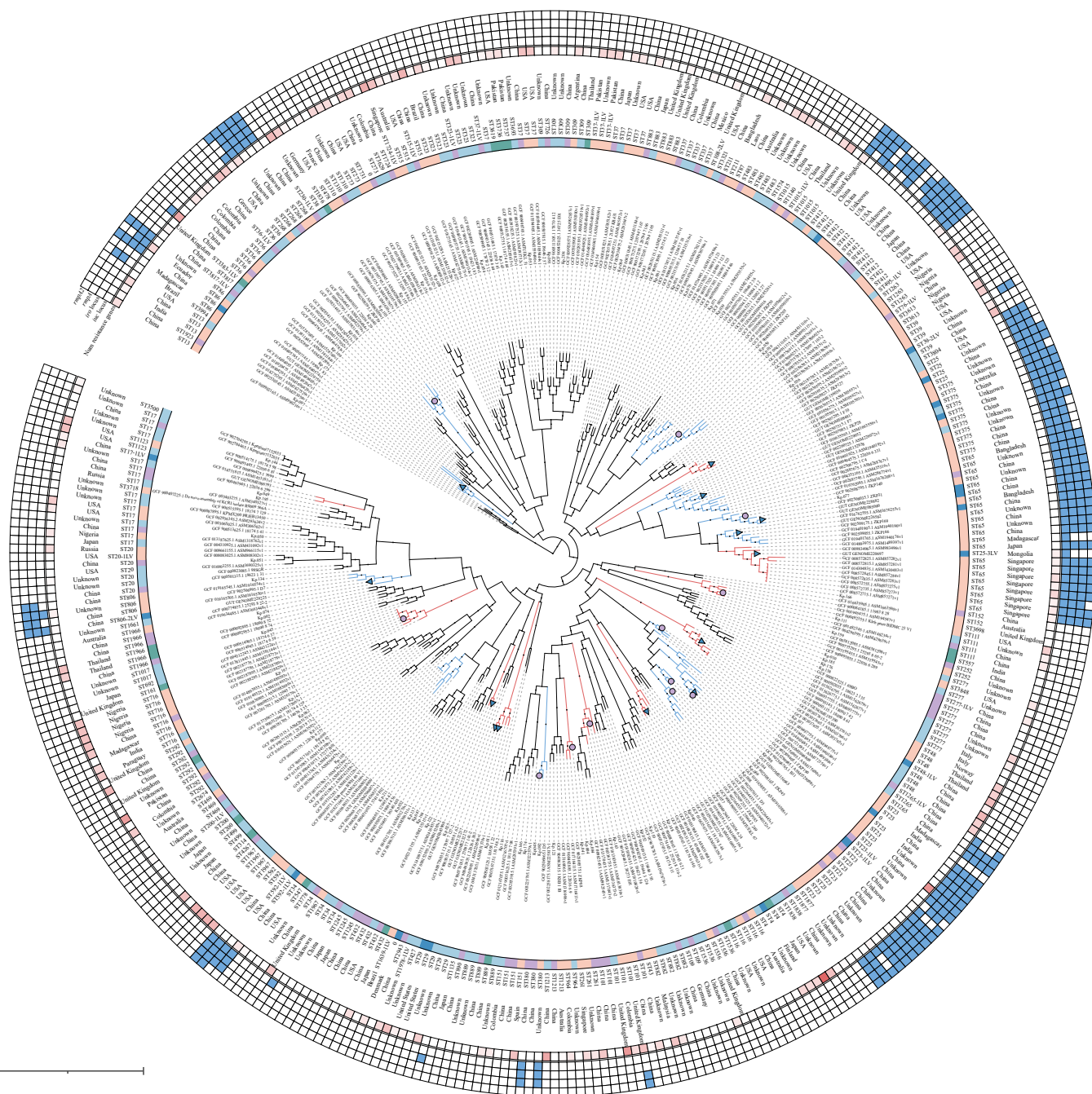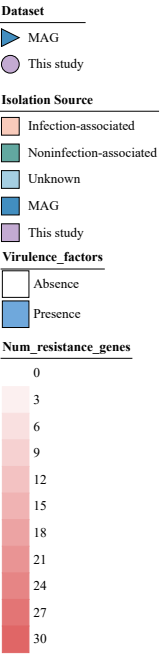

Tree scale: 0.01

Supplement: Supplemental Material [file KGMI_A_2114739_SM6750.zip › Figure S2.pdf]
